# Bibliographical Notices

**Published:** 1842-04-02

**Authors:** 


### BIBLIOGRAPHICAL NOTICES.

*First Principles of Medicine.* By ARCHIBALD BILLING, M.D., A.M., Member of the Senate of the University of London, Fellow of the Royal College of Physicians, etc. First American, from the fourth London edition. Revised and improved. Philadelphia. Lea & Blanchard, 1842.

Dr. Billing states in his preface to the third edition, that the first edition which appeared without herald of preface or advertisement, was well received, and in the present one he has taken pains to make the work more acceptable to student and physician.

One of his objects is to remove the confusion in medicine.

"It does not appear to me that I used too strong an expression formerly in speaking of the confusion which has existed in medicine; and, as an example, I need only refer to the striking fact noticed in this work, that the two words, *inflammation* and *irritation*, which are most frequently in the mouths of medical men, are up to this day perpetually used in a double or equivocal sense. Inflammation is correctly used to imply disease, and incorrectly to signify the process by which the damage done by the disease is repaired (pp. 70–72.) Irritation is perpetually incorrectly used to signify a state of disease, as it can only be correctly applied to the process whereby anything irritates, annoys, or over-excites a part: the irritant, irritating thing, whatever that be, by its operation (irritation) produces in the part morbid sensibility. One great objection to using the term iritation to imply disease, is, that irritation (the act of irritating) produces sometimes inflammation, and sometimes only morbid sensibility; but, according to the old phraseology, *irritation* produces *irritation* and inflammation, and inflammation produces sympathetic irritation and constitutional irrritation, and sympathetic irritation and constitutional irritation arise from local irritation, &c., &c. In order to avoid this equivoque, I determined, in the present edition, to adopt the term *morbid sensibility* as the name for the diseased state usually implied by irritation, and to use the word irritation only in its proper sense; and wherever the word irritation occurs in other works implying disease, it will be found that morbid sensibility may be substituted for it."

Another object is to show that medicines are not specifics, but that in different cases may act favourably in the treatment of the same diseases.

"I have shown how every medical man has his hobby to carry him to the same point, which, though he thinks it very different from his neighbour's, is as like it as one four-legged jade is to another; how one man thinks he has made a discovery that he can cure cholera with sugar of lead, and that there is nothing equal to it; whilst tartar-emetic, calomel, Epsom salts, or Glauber salts, or common salt, or mustard, or lemonade, or vinegar and water, &c., &c., will do the same thing; though none of them more quickly carry off the vomiting and purging than two of these hobbies in double harness—tartar-emetic with some neutral salt, I care little which."

As to the use of different medicines, Dr. Billing remarks aptly enough,—

"We sometimes find persons doubting the efficacy of valuable remedies from not knowing how to apply them; for instance, bark, sarsaparilla, dulcamara, logwood, carbonate of iron, arsenic, conium, digitalis, elaterium, hydrocyanic acid, and blisters; each of these has at one time or other been said to be either inert or injurious, from misapplication, though they are powerful and efficacious remedies. We every day meet with old men who from prejudice have scarcely ever used some one or other of these substances; though others, placed in an extensive field of practice, such as our hospitals, use them daily with advantage: there are even persons who have been thirty or forty years in tolerably extensive practice who have not made use of a lancet so many times.

In going round the wards of an hospital, a pupil might remark to the physician at one bed, What a small dose, and at the very next, perhaps, What a large dose, you have given; large and small being both incorrect terms when the force applied is properly adapted to the quantity of disease and state of constitution. In practice there should be no such thing as boldness or timidity: boldness is an ignorance (for we must not suppose a recklessness) of the harm which too strong means may do a fellow-creature; and timidity is an ignorance of the efficient means which remedies afford of relieving human suffering."

These extracts from the preface show the general tenor of Dr. Billing's intentions in publishing the work—to remedy the confusion existing in medicine, to define the value of pathological action and the means of modifying them by medicinal impressions. In other words, the announcement is about equivalent to a system of medicine, but, in fact, the object to which the author attaches the greatest value is simply his own hobby, and serves no real purpose—except as a frame work upon which he has attached an immense number of practical facts: the real merit of the work, and one to be fully appreciated by practitioners. They show large experience and philosophical thought on the part of the author.

Dr. Billing, while dispensing with the formality of chapters and sections, begins his book with certain preliminary observations, in the course of which he affirms that the first sound of the heart, like the second, is caused by valvular contraction. This opinion he admits to be heterodox, but thinks he has good reason for differring with the ordinary belief. As to this matter, we do not, however, agree with him; we believe that the opinion which ascribes the first sound to muscular contraction is the correct one. Animal temperature is ascribed, with very good reason, to the double process of deposition of solids from fluids, and the decarbonization of blood in the lungs.

Dr. Billing's doctrine of inflammation plays a very prominent part. The capillary arteries he believes to be weaker in action when inflamed, and that there is diminished arterial action—that is, diminished contraction. The throbbing is then the yielding to the action of the heart; of course a passive phenomenon so far as the inflamed part is concerned.

The action of the capillaries depends on nervous influence. Irritate the nerves of a part by electricity, &c., and you at last diminish the nervous power and afterwards the contractile capillary power, and inflammation of the dilated state of the capillary succeeds. Secretion is arrested in inflammation because the dilated vessels allow the blood particles to find an easier passage through the capillaries instead of the excretory ducts. If the patient be feeble without inflammation, as in hysteria, &c., the secretions may be increased in quantity. Slight irritations may increase secretion; these, however, Dr. Billing does not consider as inflammation properly speaking. Exudations of coagulable lymph, and often of pus, constitute a healthy reparative process, scarcely to be called inflammation. Morbid inflammation takes place when the nerves are inflamed, and then may be destructive of the tissues instead of restorative. A virus or morbid poison taken into the system produces a fermentation, compared by the author to a chemical movement which extends itself through the whole body.

Passing from the introductory part of the work the author proceeds to the analysis of different therapeutic agents, which, in fact, renders the volume a treatise on therapeutics; the theoretic portion being in the minds of most readers an unnecessary appendage. As a specimen of his mode of reasoning, we append a passage:

"It was long before I could account for what are called the *specific* effects of such remedies as mercury, arsenic, colchicum, &c. We can understand thus far, that the membranes, cellular tissue, skin, and parts which are very vascular, under common inflammation run a rapid course of disease, and are relievable by active antiphlogistic means; but when parts are attacked by specific inflammation, which is produced by a morbid poison, and which is slow in its progress, or when the tissue inflamed is one of dense structure with very minute capillaries, depletion, or taking off the *vis à tergo*, has little or no effect on those capillaries; and we are obliged to resort to what have been called specific medicines, such as mercury, arsenic, &c., which make them contract. Here we are supplied with analogies to help us in the prosecution of the cure of diseases with other remedies, in cases when the so-called specific either fails or disagrees: which being ascertained, the specific use of the medicine ceases—it ceases, in fact to be a specific. For instance, at one time no remedy was known except mercury against the chronic inflammation produced by the syphilitic poison. Now, taking my view of the proximate cause, we should deduce, *à priori*, that iodine might cure it, or that rigid diet and such remedies as mezereon would do so, by their effect on the capillaries; which has, in fact, been empirically proved to be the case. But it may be said, I have got no farther than the empiricism; on the contrary, I have no doubt but that arsenic would answer, but that, again, is not a fair example, as it is already used empirically in India; but iron would answer, only, not being so powerful, it would require the inconvenient adjunct of a rigid diet, as mezereon does. Again, I have no doubt that, on principle, colchicum might be substituted for mezereon; or antimony, silver, or copper, for the other chemical remedies: gold has been tried, and found to succeed. But though it be useful to have other means, when we cannot supply the ordinary one, we need not resort to a hatchet or a penknife to cut bread with, when there is a table-knife at hand; nor have recourse to any thing in preference to mercury for the cure of syphilis, from an apprehension that it may disagree, because in one in a thousand or a hundred cases it is found to do so. It is better to learn to modify it, by combining opium, &c., with it, to correct any inconvenience when it occurs; and when, of course, it is necessary to be able to bring analogical remedies into play.

The specific which puzzled me most and latest was sulphur for itch; but now the mystery is satisfactorily cleared, and we see why more powerful drugs taken internally could not cure it. Its cause being a parasitic animalcule, it is easily removed by rubbing on sulphur, which kills the little animal in his lair; whereas he could not be hurt by the remedies that cure those eruptions which are a disordered state of the capillaries, and which are easily affected by the remedies as they circulate through them. Therefore, as there are other substances which can kill the animalcule, though perhaps none so conveniently as sulphur (corrosive sublimate, for instance, might salivate before it could cure the itch,) one more specific is struck off our list. As for colchicum being a specific for gout or rheumatism, it is no such thing; there are several equally efficacious means of treating either. Again, there is no single specific for tic douloureux: cases have been cured with liq. arsenicalis, in which iron had failed, and *vice versa;* and I have cured a case with carbonate of iron, combined with galvanism, which I was told had held out against all the usual modes of treatment.* Tic douloureux may also be sometimes cured better by quinine, or opium with bark or quinine, than by any other medicine; sometimes mercury, &c., &c., are necessary. Bark is no longer a specific for ague; we can cure it with arsenic, and other remedies that cure neuralgia or neuritis, which ague is in fact."

The remarks on the treatment of fever, on the proper time to give stimulants after the febrile excitement of typhus has declined, are excellent. The same notice may be taken of those relative to proper delirium tremens, and to the simulated delirium tremens which follows operations, &c., requiring stimulants, instead of evacuants.

As we have already remarked, the work is one which causes thought, but consisting, as it does, mainly of detached reflections, having no other connexion than such as existed in the chain of ideas followed by the author, it must be much more useful to the practitioner who is familiar with disease than to the pupil. The author himself states that he found such to be the case with the former editions, but that the present one is intended to be equally well fitted for beginners as veterans in medicine. But in that and in some other respects authors are not the best judges of their own works.

---

*Lectures on the Diagnosis, Pathology, and Treatment of the Diseases of the Chest.* By W. W. Gerhard, M. D., Lecturer on Clinical Medicine to the University of Pennsylvania, Physician to the Philadelphia Hospital, Blockley, and to the Philadelphia Dispensary, &c. Philadelphia. Haswell & Barrington. 1842.

A portion of this collection of lectures appeared in the Medical Examiner in the years 1840–1. Others have been added, forming a general series on physical exploration and other means of general diagnosis, as well as the histo-

* Carbonate of iron had, of course, been already employed to a large amount; but the disease was kept up evidently by a torpid state of the liver, which had resisted mercury and other medicines. The cautious repeated application of galvanism to the organ, in about a week produced an abundant secretion of good bile, and improved the digestion; after which a perseverance with the iron for some time cured the neuralgia.

ry and treatment of individual diseases. Our remarks are necessarily limited to the announcement of the publication of the work.

---

*Report of the Pennsylvania Hospital for the Insane, for the year* 1841.

*Annual Report of the Massachusetts General Hospital for the year* 1841.

The Pennsylvania Hospital is the oldest institution in the country, in which regular provision was made for the treatment of insanity. From the date of admission of the first patient in 1752, to March, 1841, when persons afflicted with mental derangement ceased to be received into the hospital in the city, four thousand three hundred and sixty-six insane patients had received the benefits of its care. In March of last year, the insane patients were removed from their old quarters in the west wing of the Pennsylvania Hospital in the city, to the new hospital across the Schuylkill, about two miles west of Philadelphia. They are here established in a magnificent building, having a front of four hundred and thirty-six feet, situated in an enclosure of a hundred and eleven acres, and erected from a surplus fund which has been created by a judicious management of the finances of the Pennsylvania Hospital. The new Hospital for the Insane is under the superintendence of Dr. Thomas S. Kirkbride, who resides upon the premises, and to whom is confided the sole direction of the medical, moral, and dietetic treatment of the patients. The first report of the institution from Dr. Kirkbride, which we have just received, presents a very interesting picture of the organization of the hospital, and of the excellent remedial measures therein employed. The spacious and beautiful grounds in which the institution is enclosed afford the means of employment, exercise, and amusement, which are systematically and judiciously turned to account in the management of the large class of chronic cases, beyond the reach of mere medicines. The catalogue of pleasures and comforts and mental resources which are here in every way multiplied in the attempt to win back the unfortunate insane to reason, presents a truly pleasing contrast with the barbarism which prevailed fifty years ago. Agreeable occupation of mind and body, with freedom from unnecessary restraint, is recognized as the main instrument to be relied on in restoring the great mass of what are termed chronic cases of insanity. In carrying out this plan, every facility is at command in the Pennsylvania Hospital for the Insane, and is employed with paternal kindness and watchful solicitude. We hope the time is not distant when the insane poor of our State, who are, for want of means, neglected, may enjoy some of the benefits which are at present necessarily limited to a few. The results of a single year of the new system in the Pennsylvania Hospital cannot of course be taken as a test of its advantages. A very large proportion of the cases were very unpromising—old inmates of the hospital in town, and long deemed incurable. During the year 1841, 176 patients were admitted into the institution, of whom 61 were discharged or died, and 115 remain. Of the 61, 30 were discharged cured, 5 much improved, 6 improved, 11 stationary, and 9 died. Owing to the too early removal of many patients by their friends, the reporter justly remarks that the numerical statements of admissions and discharges do not give correct data for ascertaining the *curability* of insanity. Still, the institution furnishes elements for the most important results—perhaps more favourable than any similar institution. From Dr. Kirkbride, holding as he does his appointment independent of the political changes, which sometimes disturb the physicians of our State Lunatic Asylums, we may look in time for the fruits of a long series of uninterrupted observations, which he is abundantly capable of rendering of great value.

The annual report of the Massachusetts General Hospital for 1841, represents the different departments of that well ordered institution in excellent discipline. The report of Dr. Luther V. Bell, physician and superintendant of the McLean Asylum for the Insane, offers many points of interest. Dr. Bell protests against the usual system of reporting statistics connected with institutions for the insane. We agree with him in attaching no great value to the results announced in the *annual reports* from insane hospitals, and fully admit that so many circumstances complicate the history, causes, and results of the cases, that the numerical statements of "*recovered*," "*improved*," "*much improved*," &c., really convey no accurate idea of the success of treatment. We do, nevertheless, attach value to the statistical results of a series of many years from a careful observer. The disturbing elements which complicate the facts of a single year may be corrected in calculations for a large space of time; or, at least, from a vast mass of statistics the cases of a complex nature may be so eliminated as to leave deductions of real importance. Dr. Bell, however, whose opinions are entitled to great weight, says:—"I made the attempt to give a general return of the varied circircumstances relating to the cases for the first twenty years of the institution. After laboring with the amplest records before me for many weeks, the project was abandoned as neither capable of an accuracy to render it interesting to the community, and as certainly of little value to the profession. In truth, I was apprehensive that conclusions drawn from facts so uncertain, would partake quite as much of error as of truth."

He thinks that from the loose statements now put forth, "there is infinite danger that the public mind may arrive at such views and expectations as to the curability of insanity, as will eventually react most unfavourably on our successors in these holy, though arduous avocations, if not upon ourselves."

Dr. Bell's report contains many excellent and original views on the treatment of insanity. We strongly recommend all who feel special interest in the subject to peruse it entire.

